# Endoscopic Removal of Ingested Dentures and Dental Instruments: A Retrospective Analysis

**DOI:** 10.1155/2016/3537147

**Published:** 2016-09-22

**Authors:** Ken-ichi Mizuno, Kazuya Takahashi, Kentaro Tominaga, Yuki Nishigaki, Hiroki Sato, Satoshi Ikarashi, Kazunao Hayashi, Takashi Yamamoto, Yutaka Honda, Satoru Hashimoto, Kenya Kamimura, Manabu Takeuchi, Junji Yokoyama, Yuichi Sato, Masaaki Kobayashi, Shuji Terai

**Affiliations:** ^1^Division of Gastroenterology and Hepatology, Graduate School of Medical and Dental Science, Niigata University, 1-757 Asahimachi-dori, Chuo-ku, Niigata 951-8520, Japan; ^2^Department of Internal Medicine, Kameda Daiichi Hospital, 2-5-22 Nishimachi, Konan-ku, Niigata 950-0165, Japan; ^3^Department of Gastroenterology and Hepatology, Nagaoka Red Cross Hospital, 2-297-1 Senshu, Nagaoka 940-2085, Japan; ^4^Department of Gastroenterology and Hepatology, Uonuma Institute of Community Medicine, Niigata University Medical and Dental Hospital, 4132 Urasa, Minamiuonuma 949-7302, Japan

## Abstract

*Background*. Dentures and dental instruments are frequently encountered ingested foreign bodies. The aim of the present study was to assess the safety and efficacy of endoscopically removing ingested dental objects.* Methods*. Twenty-nine consecutive patients with 29 dental objects who were treated at the Niigata University Medical and Dental Hospital from August 2009 to December 2015 were retrospectively reviewed. Characteristics of the patients and the ingested dental objects, the clinical features and findings of radiological imaging tests, and outcomes of endoscopic removal were analyzed.* Results*. Patients' mean age was 62.9 ± 21.0 years. The ingested dental objects included 23 dentures (13 crowns, 4 bridges, 4 partial dentures, and 2 other dentures) and 6 dental instruments. Twenty-seven upper gastrointestinal endoscopies and 2 colonoscopies were performed, and their success rates were 92.6% and 100%, respectively. There were 2 cases of removal failure; one case involved an impacted partial denture in the cervical esophagus, and this case required surgical removal.* Conclusions*. Endoscopic removal of ingested dentures and dental instruments is associated with a favorable success rate and acceptable complications. The immediate intervention and appropriate selection of devices are essential for managing ingested dental objects.

## 1. Introduction

Foreign body ingestion is one of the most common problems for gastroenterologists in terms of performing emergency endoscopy. Most ingested bodies pass through the gastrointestinal (GI) tract successfully without requiring intervention [1]. However, sharp objects such as fish bones, medication blister packs, pins, bottle caps, and razor blades increase the risk of GI perforation [1–5]. Most foreign body ingestion occurs in children and adults with a psychiatric disorder, alcohol intoxication, developmental delay, and neurological disorders with a gag reflex impairment (e.g., Parkinson's disease, poststroke, and dementia). However, foreign body ingestion also occurs in people without these underlying conditions.

Denture ingestion is an important issue in dentistry. Most of these cases occur in elderly people because of their reduced sensation of oral mucosa and poor motor control of the laryngopharynx [6]. Moreover, the accidental ingestion of dentures and dental instruments during dental treatment procedures can occur in any patient. These dental objects have partially sharp parts; thus, there is a risk of perforation when they are ingested. Therefore, endoscopic removal of the foreign body is recommended as the initial choice of treatment because it is less invasive [7]. There are many previous reports on cases of dental object ingestion and their management [6, 8–22]. However, few reports have discussed removing them endoscopically. Therefore, the aim of the present study was to retrospectively assess the safety and efficacy of endoscopically removing ingested dentures and dental instruments.

## 2. Materials and Methods

Twenty-nine consecutive patients with 29 ingested dental objects who were treated at the Niigata University Medical and Dental Hospital from August 2009 to December 2015 were retrospectively reviewed. Dental objects were defined as dentures and dental instruments in this study. We included patients who were treated at our hospital and referral patients. Characteristics of the patients and the ingested dental objects, the clinical features and findings of radiological imaging tests, and outcomes of endoscopic removal were assessed. Written informed consent to undergo endoscopy and participate in this study was obtained from all the patients.

### 2.1. Types of Dental Objects

In this study, dentures were divided into four major types: a crown, bridge, partial denture, and other (e.g., a metal core and broken clasps). In addition, a foreign body in this study included the instrument used for dental treatment.

### 2.2. Endoscopic Removal Procedure

Endoscopic removal was performed using a single-channel GI endoscope (Olympus GIF type Q260, GIF type Q260JI, or CF type PCF-Q260JI; Olympus Medical Systems, Co., Ltd., Tokyo, Japan) with a vital sign monitor in the emergency room or in the endoscopic procedure room at our hospital. When there was a need to secure the field of view or prevent mucosal injury by the foreign body during retrieval, a distal attachment (D-206-02 or D-201-11804, Olympus Medical Systems, Co., Ltd.) was used. Grasping forceps (FG-42L-1, FG-47L-1, or FG 48L-1; Olympus Medical Systems, Co., Ltd.) or a retrieval net (00711187, Olympus) was used as a retrieval device. Intravenous midazolam was administered during the procedure if the patient was anxious or had pain. Carbon dioxide insufflation was used instead of room air when there was a risk of perforation.

### 2.3. Statistical Analysis

All variables in this study were analyzed using SPSS, version 17 software (SPSS Japan Inc., Tokyo, Japan). Variables between the two groups were analyzed using an independent Student's *t*-test or the Mann–Whitney *U* test. A *χ* ^2^ test and Fisher exact test were performed to analyze categorical variables. All tests of significance were two-tailed, and *p* values < 0.05 were considered statistically significant.

## 3. Results

### 3.1. Characteristics of the Patients and the Ingested Dental Objects

Twenty-nine consecutive patients with 29 ingested dentures and dental instruments underwent endoscopy. Patients' mean age was 62.9 ± 21.0 years (range 6–92 years), with a male : female ratio of 2.6 : 1.0 (21/8). Characteristics of the patients are summarized in Table 1. Regarding the trigger of dental object ingestion, 19 cases were due to iatrogenic causes (15 dental treatment procedures and 6 intratracheal intubations). There were no significant relationships between the triggers and patients' characteristics: age and the sex ratio. Five patients complained of some kind of symptom on arrival. Among the patients with a symptom, the locations of the foreign body were as follows: 1 at the esophageal entrance, 2 in the esophagus, 1 in the stomach, and 1 in the duodenum.

Ingested dental objects included 23 dentures and 6 dental instruments (Table 2). The symptomatic patients only included those with dentures. The types of dentures in these patients were as follows: 3 partial dentures, 1 bridge, and 1 fractured clasp. No symptomatic patients had a crown. All dental instruments were ingested accidentally during the dental procedure. All patients underwent plain radiography before endoscopy. With the exception of 1 case with radiolucent objects, 28 ingested objects were detected by plain radiography and 3 patients underwent computed tomography to confirm the location of the foreign objects and evaluate the injury. The patient with a radiolucent object (a temporary plastic crown) underwent endoscopy without radiological examination.

### 3.2. Endoscopic Removal Procedure

**Figure 1:**
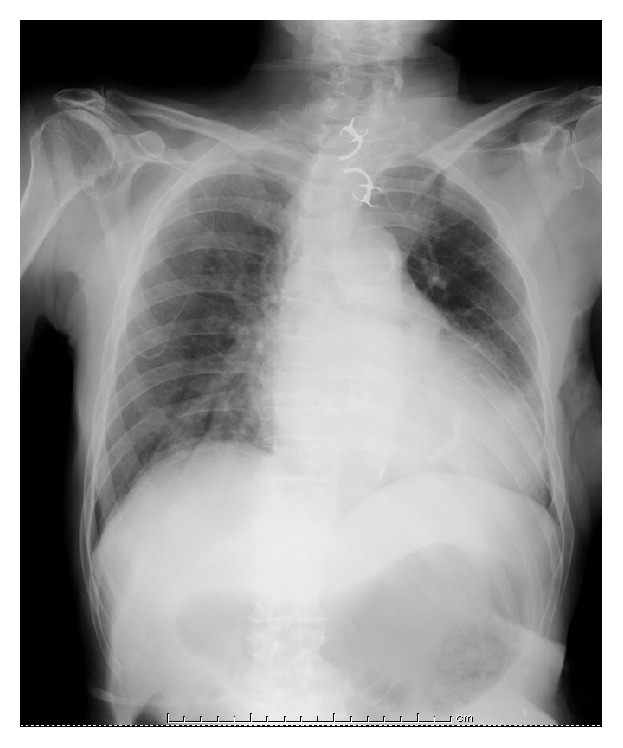
Plain chest radiography showing the ingested partial denture in the cervical esophagus.

In this study, 27 upper GI endoscopies and 2 colonoscopies were performed, and their success rates were 92.6% and 100%, respectively (Table 3). Retrieval devices were used in 26 cases. The relationship between the ingested objects and the retrieval devices is summarized in Table 4. Complications occurred in 5 patients. All complications were slight mucosal damage to the GI tract. There were no severe complications such as perforation. There were 2 cases (1 crown and 1 partial denture) of removal failure. In the case with a crown, we could not detect it by endoscopy, and plain radiography showed that it had moved into the jejunum. This patient was followed up by plain radiography, and the crown was detected in the cecum 1 week later; the patient passed the crown 51 days later. The other case of failure had an impacted partial denture in the cervical esophagus. The partial denture was equipped with clasps on both sides, measuring 57 mm by 20 mm (Figure 1). Using grasping forceps, we attempted to retrieve it endoscopically. However, it was firmly embedded in the esophageal wall. In this case, the risk of perforation was high, so surgical removal was the only possible treatment. The partial denture was successfully removed by cervical incision; the patient recovered uneventfully and was discharged on the thirteenth postoperative day.

## 4. Discussion

The present study retrospectively analyzed the endoscopic removal of dentures and dental instruments in consecutive cases for about 5 years. The inadvertent swallowing of dentures is not a rare incident in dentistry. Many previous investigators have reported it in case reports [6, 8–22]. However, the safety and efficacy of endoscopic removal of dentures and dental instruments have not been discussed thoroughly. Our study is the first to focus on the clinical practice of endoscopically removing foreign dental bodies.

Our hospital provides dentistry; hence, the cases of ingested dental objects were referred to us directly. When accidental ingestion occurs during dental treatment, dentists must perform radiography and then consult a gastroenterologist or otolaryngologist immediately according to our hospital's protocol. Among the cases of accidental ingestion that occurred in our hospital, the mean durations from the occurrence of accidental ingestion to radiography and endoscopy were 38 ± 16 min (range 20–60 min) and 120 ± 50 min (range 60–190), respectively. To achieve favorable outcomes in cases of accidental ingestion, immediate action by the dentist is essential.

**Figure 2:**
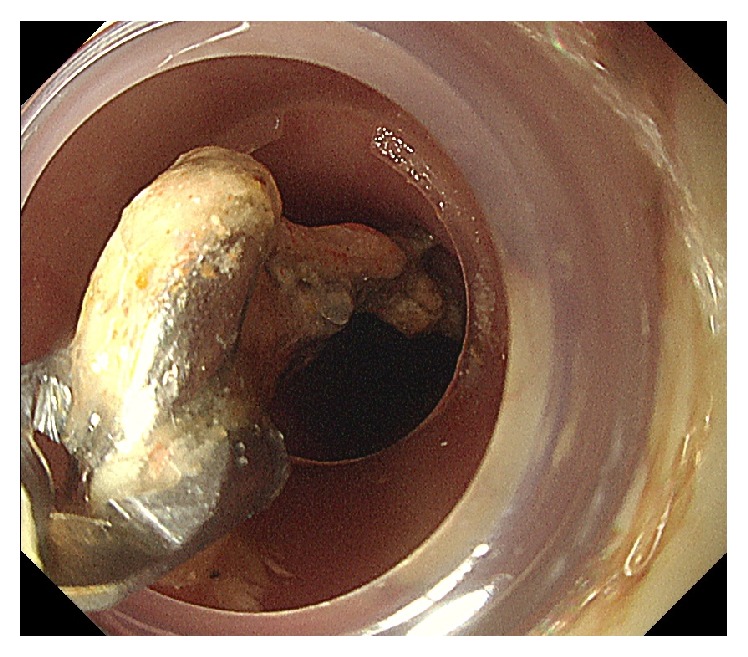
Retrieval of the partial denture using grasping forceps and a distal attachment.

Complications of endoscopic removal such as tears and perforations of the GI tract are also important issues. In our study, there were no severe complications; furthermore, slight mucosal damage occurred in 5 patients. Among these patients, the ingested dental objects included 3 bridges, 1 partial denture, and 1 fractured clasp. This indicates that the risk of mucosal injury is associated with the size of the foreign body, because crowns and dental instruments are generally smaller than bridges and partial dentures. In addition, there were no cases of injury among these aforementioned patients. To decrease the rate of complications, it is presumed that the choice of distal attachment is important. Distal attachments were used in 25 patients during endoscopic removal in this study [23, 24]. Dentures with clasps or interproximal extensions may cause injury, especially in a narrow segment [25]. When the end of the sharp part points toward the proximal side, the risk of injury during the retrieval procedure is increased. One of the distal attachments used in this study (D-2060-2, Olympus Medical Systems, Co., Ltd.) was developed for endoscopic mucosal resection using a cap-fitted endoscope (EMRC) [26], and it is 18 mm in diameter (Figure 2). Therefore, this distal attachment provides a protective cover from the sharp parts and a better visual field. To remove partial dentures, we only used grasping forceps. The retrieval net is an effective device for large and slippery foreign bodies. However, when foreign bodies have sharp parts, their sharp parts may stick out through the mess of the retrieval net and thus injure GI tracts. Therefore, the choice of retrieval devices requires attention, depending on the shape of the foreign body [27].

In the current study, there were 1 case with a crown and 1 case with a metal core detected in the cecum. The case with a crown that was conservatively followed up after removal failure showed prolonged stagnation in the cecum for more than 1 month. According to previous reports, there have been cases of colorectal impaction and perforation. Therefore, when the foreign body fails to resolve on its own, endoscopic removal should be considered [7].

The limitation of this study was its single-center, retrospective design. To determine the risk of endoscopic removal-associated complications for dental objects, large, prospective, multicenter studies are needed.

## 5. Conclusions

Endoscopic removal of ingested dentures and dental instruments is associated with a favorable success rate and acceptable complications. The immediate intervention and appropriate selection of devices are essential for managing ingested dental objects.

## Figures and Tables

**Table 1:** Patients' characteristics and clinical features.

Patients (*n*)	29
Sex, male/female	21/8
Age (years), mean (range)	68.4 (6–92)
Triggers of dental object ingestion (*n*)	
Accidental swallowing in daily life	10 (34.5%)
Dental treatment procedure	15 (51.7%)
Intratracheal intubation	4 (13.8%)
Places of occurrence (*n*)	
Our hospital	15 (51.7%)
Another hospital or clinic	8 (34.5%)
Other	6 (20.7%)
Symptoms on arrival	
Discomfort in the throat	3 (10.3%)
Pain in the throat	1 (3.4%)
Dyspnea	1 (3.4%)
None	24 (82.8%)

**Table 2:** Ingested dental objects.

Types of ingested objects (*n*)	
Dentures	
Crown	13 (44.8%)
Bridge	4 (13.8%)
Partial denture	4 (13.8%)
Metal core	1 (3.4%)
Fractured clasp	1 (3.4%)
Dental instrument	
Rubber cup (latch type)	2 (6.9%)
Dental scaler	1 (3.4%)
Dental drill bur	1 (3.4%)
Dental reamer	1 (3.4%)
Orthodontic wire	1 (3.4%)
Radiological imaging	
Plain radiography (*n*)	
Radiopaque objects	28 (96.6%)
Radiolucent objects	1 (3.4%)
Computed tomography (*n*)	3 (6.9%)
Locations detected on plain radiography	
Pharynx-esophageal entrance	2 (7.1%)
Esophagus	6 (21.4%)
Stomach	12 (42.9%)
Duodenum	4 (14.3%)
Jejunum	1 (3.6%)
Colon (cecum)	2 (7.1%)

**Table 3:** Outcomes of the endoscopic removal procedure.

Successful removal (*n*)	27/29 (93.1%)
Upper GI endoscopy	25/27 (92.6%)
Colonoscopy	2/2 (100%)
Procedure time (min), mean (range)	11 (3–30)
Type of devices used for retrieval (*n*)	
Grasping forceps	19 (67.9%)
Retrieval net	8 (28.5%)
Endoscopic suction^*∗*^	1 (3.6%)
Type of anesthesia (*n*)	
General anesthesia	4 (13.8%)
Intravenous anesthesia	10 (34.5%)
None	15 (51.7%)
Complications (*n*)	
Slight mucosal injury^*∗∗*^	5 (17.2%)
Causes of failure (*n*)	
Detection	1
Immovability	1

GI: gastrointestinal.

^*∗*^The object was pulled inside of a distal attachment by endoscopic suction.

^*∗∗*^The injury was monitored without therapy.

**Table 4:** Relationship between the type of ingested objects and the retrieval devices.

	Grasping forceps	Retrieval net	Endoscopic suction^*∗*^
Crown	6	6	
Bridge	2	2	
Partial denture	4		
Metal core	1		
Fractured claps	1		
Rubber cup (latch type)	2		
Dental scaler	1		
Dental drill bur	1		
Dental reamer	1		
Orthodontic wire			1

^*∗*^The object was pulled inside of a distal attachment by endoscopic suction.

## References

[1] Carp L. (1927). Foreign bodies in the intestine. *Annals of Surgery*.

[2] Weiland S. T., Schurr M. J. (2002). Conservative management of ingested foreign bodies. *Journal of Gastrointestinal Surgery*.

[3] Selivanov V., Sheldon G. F., Cello J. P., Crass R. A. (1984). Management of foreign body ingestion. *Annals of Surgery*.

[4] Newell K. J., Taylor B., Walton J. C., Tweedie E. J. (2000). Plastic bread-bag clips in the gastrointestinal tract: report of 5 cases and review of the literature. *Canadian Medical Association Journal*.

[5] Yamada T., Sato H., Seki M., Kitagawa S., Nakagawa M., Shimazaki H. (1996). Successful salvage of aortoesophageal fistula caused by a fish bone. *Annals of Thoracic Surgery*.

[6] Toshima T., Morita M., Sadanaga N. (2011). Surgical removal of a denture with sharp clasps impacted in the cervicothoracic esophagus: report of three cases. *Surgery Today*.

[7] Ikenberry S. O., Jue T. L., Anderson M. A. (2011). Management of ingested foreign bodies and food impactions. *Gastrointestinal Endoscopy*.

[8] Cleator I. G., Christie J. (1973). An unusual case of swallowed dental plate and perforation of the sigmoid colon. *British Journal of Surgery*.

[9] Segall M. M., Klein S. N., Bradley G. T. (1983). Colonoscopic extraction of dentures. *Gastrointestinal Endoscopy*.

[10] Price W. A., Giannini A. J. (1984). Attempted suicide by ingestion of dentures. *Journal of Clinical Psychiatry*.

[11] Dunn J. R. (1996). Patient swallows removable partial denture: a clinical report. *The Journal of Prosthetic Dentistry*.

[12] Abdullah B. J. J., Lee K. T., Mahadevan J., Jalaludin A. (1998). Dental prosthesis ingested and impacted in the esophagus and orolaryngopharynx. *Journal of Otolaryngology*.

[13] Nwaorgu O. G., Onakoya P. A., Sogebi O. A., Kokong D. D., Dosumu O. O. (2004). Esophageal impacted dentures. *Journal of the National Medical Association*.

[14] Chua Y. K. D., See J. Y., Ti T. K. (2006). Oesophageal-impacted denture requiring open surgery. *Singapore Medical Journal*.

[15] Campos M. S., Nunes F. D., Godoy R. S., Rodrigues L., Shinohara E. H. (2010). Removal of a partial denture from the esophagus with the aid of an endoscope. *The International Journal of Prosthodontics*.

[16] Abe K., Miki A., Okamura T. (2014). Endoscopic removal of a denture with clasps impacted in the ileocecum. *Clinical Journal of Gastroenterology*.

[17] Kuo S.-C., Chen Y.-L. (2008). Accidental swallowing of an endodontic file. *International Endodontic Journal*.

[18] Parolia A., Kamath M., Kundubala M., Manuel T. S., Mohan M. (2009). Management of foreign body aspiration or ingestion in dentistry. *Kathmandu University Medical Journal*.

[19] de Souza J. G. O., Schuldt Filho G., Pereira Neto A. R. L., Lyra H. F., Bianchini M. A., Cardoso A. C. (2012). Accident in implant dentistry: involuntary screwdriver ingestion during surgical procedure. A clinical report. *Journal of Prosthodontics*.

[20] Jain A., Baliga S. D. (2014). Accidental implant screwdriver ingestion: a rare complication during implant placement. *Journal of Dentistry*.

[21] Pull Ter Gunne L., Wismeijer D. (2014). Accidental ingestion of an untethered instrument during implant surgery. *The International Journal of Prosthodontics*.

[22] Vincent M., Vergnon J.-M. (2016). Foreign body of dental origin: how to retrieve the dentist's drill?. *Revue des Maladies Respiratoires*.

[23] Ginsberg G. G. (1995). Management of ingested foreign objects and food bolus impactions. *Gastrointestinal Endoscopy*.

[24] Smith M. T., Wong R. K. (2007). Foreign bodies. *Gastrointestinal Endoscopy Clinics of North America*.

[25] Gallas M., Blanco M., Martinez-Ares D., Rivo E., García-Gontán E., Cañizares M. (2012). Unnoticed swallowing of a unilateral removable partial denture. *Gerodontology*.

[26] Inoue H., Kawano T., Tani M., Takeshita K., Iwai T. (1999). Endoscopic mucosal resection using a cap: techniques for use and preventing perforation. *Canadian Journal of Gastroenterology*.

[27] Jeen Y. T., Chun H. J., Song C. W. (2001). Endoscopic removal of sharp foreign bodies impacted in the esophagus. *Endoscopy*.

